# Reasons for low utilization of intrauterine device utilisation amongst short term contraceptive users in Hossana town, Southern Ethiopia: a qualitative study

**DOI:** 10.1186/s12905-022-01611-6

**Published:** 2022-02-04

**Authors:** Demelash Woldeyohannes, Abinet Arega, Lillian Mwanri

**Affiliations:** 1 School of Public Health, Collage of Medicine and Health Science, Wachemo University, Hossana, Ethiopia; 2 College of Medicine and Public Health, Flinders University, Health Sciences Building, Sturt Road, Bedford Park, Adelaide, SA 5001 Australia

**Keywords:** Intrauterine devices (IUDS), Contraceptives, Qualitative study, Ethiopia

## Abstract

**Background:**

Intrauterine devices (IUDs) are one of the long-acting, safe and effective methods of contraception in women across the world. However, this method is underutilised in many countries, including Ethiopia. Several quantitative studies have been used to address this problem and generated a list of factors associated with this problem. However, this list lacks detailed and local contexts that are necessary to inform local solutions. The current study uses a qualitative method to explore determinants of IUDs underutilization among short term modern contraceptive users from the maternal health services in the study setting. The use of a qualitative study design is necessary to obtain and rich contextual details that can inform the development of locally appropriate strategies to increase the IUDs uptake in the study area and improve women’s reproductive health outcomes.

**Method:**

A qualitative study was conducted in Hossana town public health facilities, Southern Ethiopia from November 1–30, 2019. A total of thirteen in-depth interviews were conducted including with: 11 short term contraceptive users, one health centre head and one health extension worker. The interview guide comprised semi-structured questions. Interviews were audio recorded, transcribed and collected data analysed thematically.

**Result:**

The main key determinants of IUDs service underutilisation were identified from participants’ narratives, including: (1) poor knowledge about the benefits of IUDs, (2) insufficient counselling and ineffective delivery of health information to aid women in decision making, (3) the absence of trained health personals, and shortage of supplies.

**Conclusion:**

Results indicate that the poor utilisation of IUDs services is determined by both the service provider and the consumer related factors. Poor knowledge of short term users of contraception is a critical factor because without knowledge, clients may not use the available services effectively. The shortage of necessary supplies, poor provider–client relationships, and poor counselling by service providers are also service factors that act as barriers to uptake of IUDs. Efforts should be made to increase IUDs utilization by focusing on educating women about the importance of IUDs, improving counselling of mothers and strengthening the health systems, including allocating more resources to increase access to IUDs among the service users.

## Introduction

Globally, the total fertility rate ranges from 1.7 children per woman in the developed world to 4.6 in underdeveloped world. With the total fertility rate of 4.5 children per woman, Ethiopia is one among countries with highest total fertility rates in the world. In order for the total fertility rates to fall to low levels worldwide, highly effective, long-acting methods like IUD in less developed countries, including in Ethiopia [1–3].

Across the world in 2012, four in ten pregnancies were unintended, of which 50% ended in abortions and 13% in miscarriages [4]. Unintended pregnancies, for which nearly half of them are due to inconsistent or incorrect contraceptive use including IUDs, are an important public health concern due to their association with poor social and health outcomes for both mothers and children [5–7]. Moreover, evidence exists that effective utilisation of IUDs reduces abortions, minimise unintended pregnancies, and lower the incidence of maternal mortality and morbidity rates. Globally, it has been established that, if all women with unmet needs for contraceptives used IUDs, it would prevent 24 million abortions, six million miscarriages, 70,000 maternal deaths and 500,000 neonatal deaths would be prevented [8, 9].

Worldwide, IUDs are a recognised to be a modern long-term reversible contraceptive method which is suitable to prevent unwanted pregnancies for women of all reproductive ages [10, 11].

From 2005 to 2019 in Ethiopia, the utilisation of all types of contraception except IUDs, have increased from 14 to 41% respectively, in all women of reproductive age. The most popular contraceptive method was injectable and the least utilised method was IUDs (2%) [12].

Several studies have indicated that a number factors account for the poor utilisation of IUDs. A study conducted in Ethiopia showed that women’s perceptions and poor knowledge about IUDs were the main determinants of the low utilisation of IUDs [13–16]. Among the factors, comprehensive counselling about contraceptive usage, awareness creation about IUDs at the health facility level and focused messages to dispel misconceptions in the communities were identified as necessary strategies to increase IUDs utilisation [17]. These factors are related to healthcare provider characteristics, health system and individual user factors [18, 19]. This current study explores and seeks to understand the contextual determinants of IUDs underutilisation among short term modern contraceptive users in Hossana town public health facilities, Southern Ethiopia.

## Method

### Study setting

The study was conducted in Hossana town, the capital of Hadiya Zone, located 235 km far from Addis Ababa, the capital city of Ethiopia, and 194 km far from the regional city, Hawassa. According to the 2015/2016 Hossana town report, the town had a total population of 100,501, of which 50.73% were males and 49.27% were females. This study setting was purposively selected in consultation with regional health bureaus based on the low utilization of IUD as the main criteria.

### Study design and participants

Between 1st and 30th November 2019, a qualitative inquiry using in-depth interviews was conducted with 13 participants. These comprised: 11 short term modern contraceptive service users from three health centers and two key informant Interviewees (KIIs) one of whom was the head of the health center and another, the urban health extension worker. These health centres have the similar characteristics including socio-demographic, sociocultural and low utilization of IUDs. The purposively selected short term contraceptive users were interviewed in the health center during their visits for short term family planning methods. Interviewees were invited from this set of participants until the saturation was reached including when there were no new ideas generated. The two key informants were selected based on their role in family planning service provision in the maternal health service program in Hossana town.

### Data collection procedure

The semi structured open ended interview collection guide was developed after defining the research objectives and reviewing relevant literatures. The tool was first developed in English then translated into a local language, Amharic. A pre-testing of the tool was carried out in Fonko health centre and Balesa health centre which are services outside of the study setting, Hossana town. Interviews were tape recorded and field notes taken. Interviews were conducted by the principal investigator (DW) and a trained data collector who was familiar with the qualitative data collection method and family planning services. The interviews lasted an average of 45 min and were held in a free room near the family planning departments. The key informants were interviewed in their offices as these were quiet and private.

### Data analysis procedure

Data were transcribed verbatim and translated into English language. The two researchers (DW and AA who speak both English and Amharic fluently) critically read and reviewed all the transcripts for accuracy and completeness and analysed the data using a thematic analysis (TA) method for identifying and analysing patterns of meaning in qualitative dataset [31]. For the TA, an initial coding structure was developed through open coding method. In order to ensure the relevance and appropriateness of the coding structure, one coder (AA) conducted open coding of emergent themes associated with the study objectives. The second coder (DW) independently coded the same transcripts. Finally, the two coders reviewed the coded transcripts to reach consensus on coding structure and consistent code definitions which was used to code all transcripts and in organising themes and sub-themes, supported with key quotes and narrations.

### Data quality management and ethical considerations

To ensure the quality of the data, data collectors were recruited from study communities and were trained. The appropriateness and relevance of the guides were ensured through expert reviews and pre-testing. To enhance inter-coder reliability, the researchers independently applied the code guide to a selected transcript, and then reviewed and resolved any differences. Reports of quotations for selected codes were generated and the researchers prepared content summaries of thematic findings.

The Ethics approval was obtained from Wachemo University College of Medicine and Health Science institutional Review Board, and the research was conducted in accordance with the ethical principles. Permissions were obtained from relevant offices. Informed Written consent was obtained from all participants after explaining the study purpose and data collection procedures. All participants were anonymised for privacy purposes.

## Results

### Socio demographic characteristics of the study participants

A total of 13 study participants were included in the study: including eleven short term contraceptive users, one head of the health centre and one health extension workers/community health worker/. Participants’ ages ranged between 19 and 32 years old. All 11 short term contraceptive users had at least one child, were married house wives, and had the following religious backgrounds: five were protestant, five were Orthodox and one was a Muslim. Their education backgrounds included: six being illiterate with no formal education (could not write and read), three had finished secondary school and two had education above secondary schools. Five service users used injectable contraceptives, four used pills and two used implants. Of the two key informants interviewees (KII), who from here on will be referred to as service providers, were a male and female, both with education above secondary school level. Of these, the male service provider was the head of health centre and the female service provider the health extension worker (Table 1). Data analysis led to the emergence of three themes as presented and detailed below:

**Table 1:** Socio demographic characteristic for short term contraceptive user women

Characteristics	Number
*Age group for mother*	
19–24	5
25–32	6
*Marital status*	
Married	11
*Religion*	
Protestant	5
Orthodox	5
Muslim	1
*Number children*	
1–2	2
3–4	5
≥ 5	4
*Educational background*	
No formal education	6
Secondary school	3
Above secondary schools	2

### Participants’ poor knowledge about the IUDs’ benefits and source of information

Women seemed to know the benefits of short term contraceptives such as pills, injectables and condoms, but had poor knowledge about IUDs and their benefits. Service users indicated these benefits of these non-IUDs to include both financial health and other benefits. The main source of information was described to be the social media and the urban health extension worker located in their communities. These assertions are supported by narratives of service users as exemplified below:

I knew most of the contraceptive methods by name including pills, injectable, condoms, and a contraceptive which are inserted on the upper arms. And I knew well about the benefits of these contraceptives except IUDs. These short term contraceptives have benefits for child spacing, for child limiting and health of the mother and her child, it saves the unnecessary expenses of the households and the country. I heard this information from different sources including TV, Radio and Brushers from Health centre and NGOs. Additionally, urban health extension workers (community workers) gave me some information about contraceptives methods (short term contraceptive user 1, female aged 29 years)

Using short term contraceptive method has several benefits; among those benefits, it helps me to have a smaller family size. If we have a small family size we would teach and rear our children properly without financial constraint. Another benefit of using these contraceptives is to have sexual intercourse without any stress that means we will not worry about whether I will be pregnant or not. Now, I don’t want to have more than two children in my lifetime. (Short term contraceptive user 2, female aged 21 years)

Although women demonstrated to have knowledge about the benefits of the short term contraception use, there was also negative perceptions of how these contraceptives did work. Furthermore, perceived negatives of health outcomes when IUDs were used including development of obesity seemed to be deterrent to women using these. The source of these misinformation and misunderstanding seemed to originate from within the communities, including from their friends and media.

I heard about IUDs from Television as it’s inserted into Womb. It is only removed by health professionals when I want to remove it. My friend used IUDs and she complained as she faced health problem and she became obese “bokach’ means ‘being fat’ then she discontinued. And she advised me not to use IUDs but use an injectable contraceptive method instead. (Short term contraceptive user 3, female aged 23 years)

The negative perceptions about the complications of IUDs expressed by service users were also a common knowledge by service providers including by the head of Hossana health centre, who associated the low utilization of IUDs with the lack of awareness and knowledge about the benefits of IUDs. In his own words, He stated:

In our health centre, the short term contraceptive utilization coverage is greater than 90% except for IUDs, the reasons for low utilization of IUDs is the lack of awareness about IUDs, lack of knowledge about benefits of IUDs, misconception about IUDs and fear of side effects are the reasons for not utilization of IUDs (service provider 1, male aged 26)

On the same vein, the urban health extension worker (community health worker) had views supportive of the head of health centre’s assertion that poor perceptions about the effectiveness of IUDs had deterrent effects on IUDs utilisation in Hossana. As she had worked as urban health extension worker /community health workers/ for seven years, she acknowledged a gap in service provision and the lack of collaborative effort among different stakeholders to address these issues. The lack of effective service provision was understood to have resulted in in low utilisation and poor perceptions of the benefits of IUDs among the service user. These assertions are supported by her statements below.

…only our effort is not enough to create sufficient awareness and knowledge about IUDs. I think it good to integrate our effort with different stakeholders at the community. We are not delivering strong health education about IUDs as expected from us at the community level; hence the level of awareness and knowledge of the community about IUDs is very low (service provider 1, male aged 26)

### Myths and misinformation for effective decision making about the use and benefits of IUDs

Adequate counselling is necessary as an effective tool to aid women’s decision about types and effective use of and any contraceptive method. The lack of effective counselling and inadequate provision of appropriate public information for the women to use may also be the cause of the shortfall in IUDs utilisation. The assertions below further reinforce how the local health services are poorly equipped in helping the service users to increase IUDs utilisation.

I am coming to this health centre every three months to receive the injectable contraceptive/Dipoprivara/ method but I haven’t received any counselling about the benefits of contraceptive you are asking /IUDs/. After they gave me the injection, they give me an appointment to come back after three months on appointment date (Short term contraceptive user 4, female aged 23 years)

Poor engagement of service users by service providers seemed to further affect women’s health resulting in discontinuation of the service. This is exemplified by the woman below who had been using IUDs for three months. Poor service delivery was also stated as the reason to exiting the service by a woman who found private sector services to be much better. These assertions are confirmed by statement below.

I used IUDs for the last three months. After I have started using this method my behaviour was completely changed ‘yanagergnal’, ‘yabesachegnal’ means it makes me ‘talkative’ and ‘irritable’. Moreover, I feel some discomfort around my abdomen. Then I went to a clinic to get advice from Health personals, the health care provider pressured me to change another type of contraceptive methods without telling the reasons of those problems (behavioural change and discomfort around the abdomen) but I didn’t agree with health personal advice then I went to a private clinic near to my home. And the health personals in privet clinic removed IUDs and I started using the injectable contraceptive method. After starting the injectable method my behaviour and my health is improved rapidly (Short term contraceptive user 5, female aged 30 years)

Misinformation from within the community about the adverse effects of IUDs, also influenced others to not use them. As for service user 6, who is a new user of contraceptives seemed to have received, misinformation from a friend who had been on IUDs. The friends’ myths about IUDs seem to have influenced her decision against using IUDs.

I am a new user for short term contraceptive method/injectable/ and I got some information from my friend she was previously IUDs user and I had never got counselling from the health personals about benefits of IUDs. She told me to use another type of contraceptive methods, she told me as it causes infertility as well as it is not easy to remove from the womb as another contraceptive methods and it’s not comfortable at the time of sexual intercourse (Short term contraceptive user 6, female aged 31 years)

Similarly, for service user 7, aged 19 years, misinformation including the potential of IUDs to cause discomfort during sexual intercourse, led her to avoid this type of contraception. Perceived inadequacy of service provision including a lack of technical expertise in the setting was also an important factor that precludes patients from using IUDs as described below.

I decided to not use this type (IUDs) of contraceptive method because I was afraid of the side effects, it might be not comfortable to have sexual intercourse. Frequent checking of IUDs tread is boring and I have a fear of it might change the position at the time of hard works. At the time when I want to remove, I could face several problems to remove it such as the absence of trained health personals and absence necessary equipment’s to remove it. (Short term contraceptive user 7, female aged 19 years)

Similar assertions including doubts about the available expertise in the setting such as adequately trained service providers, seemed to be a significant reason for service users to lack information about the availability, benefits and further information about why women should use IUDs.

…….for your surprise, on my first visit to this Health centre, I asked the health personal about what would be better for me? But the health personals told me to use injectable contraceptive method without telling about any available contraceptives in the health centre including IUDs. (Short term contraceptive user 8, female aged 19 years)

I want to the health centre to know the types of available contraceptives to choice and I have asked one of the health officers in the health centre about currently available contraceptive methods and its benefits. He told me about all available contraceptive methods and their benefits. Then, I preferred to use Implant rather than IUDs because I scared of solid device insertion in my Womb then I think it might have health problems including genital area infection and I could bleed at the time of insertion. (Short term contraceptive user 9, female aged 23 years)

### Absence of trained health personals and shortage of supplies

Supplies of IUDs and the absence of adequately trained health personals for insertion and the removal of IUDs were among important reasons stated as a cause of IUDs underutilisation. As explained below by participant who uses pills and Implants, even when they had a desire to use IUDs, the lack of expertise in service provision as well as sufficient supply of the service, they found themselves choosing other types of contraceptive methods. These assertions are exemplified below:

I heard the benefits of IUDs from TV in my home and my neighbour told me about the benefits of IUDs then I decided to use IUDs and I want to the health centre. But they said no IUDs device and no trained health personnel for insertion of IUDs. And they told me to use another type of contraceptive method (Short term contraceptive user 10, female aged 25 years)

After I heard the benefits of IUDs from radio then I went to the health centre to use IUDs but Nurse in family planning unit says in our health centre, the IUDs insertion equipment has been stocked out before three months and the trained nurse for insertion of IUDs left to her Master's education. Hence, to not have unwanted pregnancy I decided to use short term contraceptive methods (Short term contraceptive user 11, female aged 27 years)

## Discussion

The focus of this study was to explore determinants of IUDs underutilization among short term modern contraceptive users among women in Hossana public health services, in Southern Ethiopia. The study identified three major themes related to both the health system and contraceptive users that acted as barriers to IUDs utilization, including: poor knowledge of study participants about IUDs, inadequate counseling about IUDs, absence of trained health professionals and shortage IUDs supplies. These are important findings that can inform service planning and delivery as well as for research and improvement in the body of knowledge.

It is well acknowledged that health systems in many low income countries have been inundated by poor health service delivery for many years. The 1993 World Development Report ‘Investing in Health’ advocated for more investments in the health sector in low- and middle-income countries to improve individual health outcomes and productivity and to strengthen economic growth [21]. Based on the current study finding, giving attention to health education about the benefits of IUDs to increase the knowledge level of service users to assist them to make appropriate decision regarding the IUDs. The better provision of accurate information to a broader audience including women, health workers and the community in general, would improve the reproductive health outcomes of these women, as would counter the misinformation and myths which seemed to be a significant factor in the study setting. Women who had experienced side effects with other methods seemed more open to trying a new methods including IUDs. Despite the misinformation, a few had a strong initial interest in using the IUDs method because they did appreciate it due to be a long-acting, effective, hormone-free, and would preserve normal menses. These findings are similar to the studies conducted in Ethiopia and Nigeria [22–24], and consistent with previous studies conducted in Addis Ababa, Ethiopia, which demonstrated that increased level of knowledge about IUDs and an understanding about effectiveness of long-acting contraceptives, increased the use of IUDs [13, 25].

To counter the effects of misinformation and false perceptions about IUDs, the effective counseling about available contraceptives and the provision of systematic and accurate information by service providers could be among key strategies to improving utilization of IUDs in the current study setting. While the knowledge about types of contraceptive methods was not as high among participants, the finding of the current study suggested that there were inadequate counselling services for the women about IUDs from the health personals and from health extension workers/community health workers, Improving the effectiveness of the counselling services and increasing time to discuss issues related to short term contraceptive usage including IUDs between service providers and service users could help to increase the utilization of IUDs [26, 27]. The increase in counselling service for efficient decision making among service users could help and prevent misconceptions and rumours about negative effects of IUDS that appeared to prevail in the current study setting. At the time of in-depth interview, misunderstanding about IUDs was not well-addressed by counseling and providing the contraceptives including IUDs by the health personals and community health workers. This finding is not surprising and supported by studies conducted elsewhere in poor resourced settings including in Pakistan [28–31]. The IUDs underutilization is such a public health issues of significance that requires quality counseling and follow up in order to increase utilization of IUDs [32–34] and to improve inequalities in women’s health. Health personals and health extension workers could encourage IUDs utilization by giving factual information tailored to counteract rumors and misconceptions about IUDs.

Supplies of IUDs and absence of trained health personals for insertion and removal of IUDs were among the reasons for the low utilization of IUDs. Even when the women were interested to use IUDs, the lack of the supply of these devices and the service provision by poorly trained service providers, complicated women’s understanding of the services and hindered utilisation as women would not trust the services. Shortage of supplies and the absence of trained health personals should be addressed by the concerned body to raise the utilization of IUDs [35] in Ethiopia.

### Limitation and strength of the study

This study has several limitations including small sample sizes and the focus on group of women in Hossana town in Southern Ethiopia. With the resources we had, it was not possible to include broader setting to enable comparisons of perspectives. The social desirability bias could also be considered as a limitation but we minimized this by conducting interviews using trained data collectors and by using the local language. The use of a qualitative design while can be considered as a strength due to obtaining rich and contextual perspectives, it may also limit the generalisation of the study findings to include broader audience. Regardless, the findings from this study may reflect problems that exist in Ethiopia and in similar settings in developing countries. In addition, this study has given voices to those who have expressed genuine concerns about the potential impact and barriers to underutilisation of IUDs. IUDS are known to be effective and easy to use contraceptive, and when used effectively, they can improve family planning for families and communities in developing countries.

## Conclusion

Identifying factors related to poor utilisation of IUDs is critical in informing strategies that target family planning among the specific subpopulations. Moreover, understanding different contextual factors associated with predictors of poor IUDs underutilization among participants may as well address significant public health problems such as unwanted pregnancies and maternal morbidity and mortalities. For the current study setting, the finding of this study identified the major reasons for IUDs underutilization to include poor knowledge about IUDs, inadequate counselling and inadequately informed choices of available contraceptives. Additionally, poor availability of trained health personals and shortage of supplies were additional predictors of poor utilization of IUDs. To improve IUDs utilization it is necessary to improve the way health education and counselling is provided in order to increase the awareness level of women. Providing on-job training to the health care providers may be needed to avoid the shortage of trained health care providers. These finding provide significant evidence that can be used to improve practices and policies in the discipline area.

## Data Availability

The spread sheet data supporting the findings of this is available at the hands of the corresponding author which can be delivered to the journal based on request at any time.

## Abbreviations

ANC: Antenatal care
AOR: Adjusted odd ratio
FP: Family planning
IEC: Information Education Communication
IUDs: Intrauterine device
